# Liver and Cardiac Involvement in Shwachman-Diamond Syndrome: A Literature Review

**DOI:** 10.7759/cureus.6676

**Published:** 2020-01-16

**Authors:** Odunayo S Lawal, Nimisha Mathur, Srilatha Eapi, Rupak Chowdhury, Bilal Haider Malik

**Affiliations:** 1 Pediatrics, California Institute of Behavioral Neurosciences and Psychology, Fairfield, USA; 2 Internal Medicine, California Institute of Behavioral Neurosciences and Psychology, Fairfield, USA; 3 Pathology, California Institute of Behavioral Neurosciences and Psychology, Fairfield, USA

**Keywords:** shwachman diamond, cardiomyopathy, hepatomegaly, myelodysplasia

## Abstract

Shwachman-Diamond syndrome (SDS) is an autosomal recessive inherited disease of the SBDS gene. It has multi-organ involvement but primarily affects the bone marrow and the pancreas. This disease is more commonly found in males than females, and its earliest manifestation in infancy is pancytopenia, most especially neutropenia. Our article attempts an in-depth analysis of the hepatic and cardiac association in this disease and the severity of this association.

For the purpose of this study, we engaged in an in-depth research of critically appraised literature and published articles. We searched for such articles on PubMed and Google Scholar using regular and Medical Subject Headings (MeSH) keywords. We eventually selected 32 articles from the search results and carefully read through and analyzed them. These articles showed the usual age of diagnosis of SDS to be at infancy (before age one), with a predominantly median survival age of 35 years.

All the published articles we reviewed showed some hepatic and cardiac associations with SDS, but the extent of the associations varied. Even though most hepatic involvements were found to be benign, some severe cases led to fibrosis and hepatic failure. Although there is no particular consensus as to the exact outcome of cardiac involvement, the few cases we reviewed showed that cardiac association could be a severe complication and could even be fatal. Most of the cases reported in the literature had been diagnosed at autopsy.

## Introduction and background

Shwachman-Diamond syndrome (SDS) is a genetic mutation that primarily affects the bone marrow and involves multiple organs. It is a rare condition and not much information is available on it in literature. However, researchers are making steady progress in their quest to find ways to further understand the disease, prevent complications, and better manage it appropriately. To quote William Ramsay, “Progress is made by trial and failure; the failures are generally a hundred times more numerous than the successes, yet they are usually left unchronicled”. Only a few cases have been recorded worldwide, which revealed an incidence rate of 1/77,000 and a predisposition towards males than females (1.7:1). Approximately 90% of the reported cases have been found to have a genetic mutation involving both alleles of Shwachman-Bodian-Diamond syndrome (SBDS) genes found on the long arm of chromosome 7 at position 7q11. The remaining 10% remains uncharacterized [1,2]. Even though the role of this gene is not yet fully known, literature has established that it is involved in ribosome maturation [3].

SDS is an inherited autosomal recessive disease of bone marrow failure, associated with an insufficient function of the exocrine pancreas and bone and skeletal deformities [4]. The earliest clinical manifestation of the disease usually occurs in infancy as pancytopenia with the majority being neutropenic [5]. This makes patients susceptible to infections, malabsorption, leukemia, failure to thrive, and rib cage abnormalities [6,7]. Other rare associations are cardiac pathology and severe hepatic dysfunction, although mild hepatomegaly and elevated transaminases have also been reported in the literature [8,9].

In this review, the hepatic and cardiac association is carefully researched with the aid of literature and published works. We have attempted a thorough critical appraisal and discussion to gain more insight into this disease and its associations.

## Review

Methods

We used PubMed as the primary database for finding articles on the subject. We also used Google Scholar. No systematic review guidelines such as Preferred Reporting Items for Systematic reviews and Meta-Analysis (PRISMA) were used as this was for a traditional review article. No inclusion and exclusion criteria were used. We did not focus on any specific demographic details in selecting literature for review. A set of regular keywords and Medical Subject Headings (MeSH) keywords were used, and the results were recorded, as seen in Table 1 and Table 2. 

**Table 1:** Regular keywords used in literature search

Keyword	Database	Results
Shwachman-Diamond syndrome	PubMed/Google Scholar	480/5,890
Cytopenia	PubMed	2,739
Hepatomegaly	PubMed	11,650
Myelodysplasia	PubMed	3,429
Cardiomyopathy	PubMed	121,776
Bone marrow	PubMed	86,076
Shwachman Diamond syndrome and liver	PubMed	34

**Table 2:** MeSH keywords used in literature search MeSH: Medical Subject Headings

Keyword	Database	Results
Cytopenia	PubMed	0
Cardiomyopathy	PubMed	104
Myelodysplasia	PubMed	13

Results

The keyword Shwachman-Diamond syndrome generated 480 and 5,890 articles on PubMed and Google Scholar, respectively. Other keywords were searched mainly on PubMed, with cytopenia generating 2,739, and Hepatomegaly returning 11,650 articles; myelodysplasia resulted in 3,429 articles; cardiomyopathy brought up 121,776 articles; bone marrow gave 86,076 articles, while Shwachman-Diamond syndrome and liver generated 34 articles. Of these keywords, some were searched under the Medical Subject Headings (MeSH) criteria, with cytopenia generating 0 results. Cardiomyopathy returned 104 results, and myelodysplasia generated 13.

We reviewed 32 published articles of cases from different continents (Asia, Europe, and the Americas). There were 99 cases in total with different study designs; 15 of those were case reports from 13 different articles. There was a Spanish article included in this review, and this was translated into English using Google Translate.

We noticed throughout the literature that the age of diagnosis was usually at infancy (before age one). The condition was more commonly found in males than females. The average age of survival was 35 years.

Discussion

Pathophysiology

SDS is currently recognized as the second most common disease of exocrine pancreas with cystic fibrosis being first [5]. The primary pathophysiologic mechanism involves bone-marrow failure and exocrine pancreas deficiency leading to a wide range of manifestations [10-11].

As the bone-marrow suppression usually leads to neutropenia with reduced mobility of the neutrophils, patients are susceptible to various infections ranging from systemic to cutaneous [12]. Thrombocytopenia and severe aplastic anemia are also seen in some patients. Increased levels of fetal hemoglobin with macrocytosis of the erythroid cells have also been reported, which could increase the risk of these patients developing leukemia, especially acute myeloid leukemia (AML) and myelodysplastic syndrome (MDS). These mentioned conditions have been fully described by S.Y. Ong et al. in a case reported from Asia involving a 19-year-old male patient. The progression of these marrow disorders is the main source of mortality and morbidity in such patients [13-15].

The pathophysiologic mechanism in the exocrine pancreas involves a severe reduction in the pancreatic acinar cells, which leads to various clinical gastrointestinal manifestations, ranging from being asymptomatic to severe malabsorption symptoms such as abdominal cramps, steatorrhea, and even failure to thrive. These manifestations tend to occur within the first year of life. An updated review done by Myers et al. discussed a study done by Shah and colleagues about the histologic changes in the biopsy of the gastrointestinal mucosal of 15 patients who were genetically diagnosed with SDS. More than 50% of these patients had shown various degrees of duodenal inflammation [1], which suggests that apart from exocrine pancreatic failure, these patients also showed symptoms that indicated some enteropathy components to the gastrointestinal system.

The skeletal system is also a major organ frequently involved in this disease. Some of the related features observed are growth disturbance, metaphyseal dysplasia of long bones, especially the proximal part of the femur. However, this rarely manifests in neonates, unlike the dysplasia of the short ribs with anterior ends that are cupped and flared, which is also associated with this disease and appears in the neonatal period, which causes respiratory distress [16]. Deficient chondrogenesis also affects the cartilaginous part of the respiratory system according to a published article on two case reports of an SDS patient who presented with dyspnea due to subglottic stenosis [17]. It is an extension of the skeletal system complication that is extremely rare.

Hepatic and Cardiac Association

There is more than enough evidence throughout the literature establishing the involvement of liver in this disease, ranging from an asymptomatic increase in transaminase levels, hepatomegaly, fatty infiltration of the liver to different forms of persistent chronic liver disease due to hepatic fibrosis, including cirrhosis [18,19]. In the majority of the cases reviewed, abnormal transaminase levels were detected in neonates and these returned to normal with increasing age. However, there were a few cases found with a degree of portal hepatic fibrosis and persistently elevated transaminase levels [20]. Also, there were some cases which showed subliminal liver dysfunction complicating and causing liver failure in a condition that would not have otherwise been so, such as a case of severe hepatic failure requiring transplant due to fibrosis with portal hypertension leading to hepatopulmonary syndrome in toxicity with Vitamin A [7]. This asymptomatic hepatic dysfunction can also complicate bone marrow transplant (BMT), which is a major way of managing this disease, causing nonalcoholic steatohepatitis leading to liver failure, as seen in the case report of a 35-year-old woman reported by DS Ritchie[21].

Cardiac involvement in SDS is not well documented. However, there have been some fatal cases recorded throughout the literature [22,23]. A retrospective and prospective study carried out by Ryan et al. on 17 cases, which were first analyzed retrospectively based on their data and then followed up for a period of time to document their cardiac changes using echocardiographic measures, showed that there was abnormal systolic dysfunction in 33% of these patients [8]. Atrioventricular septal defect involvement has also been reported in a patient with SDS [24]. A study conducted by Toiviainen-Salo on the myocardial function in SDS patients established that no abnormalities were found in the myocardial structure and the cardiac anatomy of these patients. However, they observed alterations in the right ventricular diastolic function at rest and a decreased left ventricular contractility during exercise [25]. All this evidence shows that there is cardiac involvement in SDS, and clinicians should pay close attention to monitoring such patients as this can lead to dire complications. A few of the articles demonstrating hepatic and cardiac involvements are shown in Table 3.

**Table 3:** Different articles highlighting the hepatic and cardiac involvements in Shwachman-Diamond syndrome SDS: Shwachman-Diamond syndrome

Author	Journal (year of publication)	Type of study	Findings
Camacho, et al. [18]	World Journal of Clinical Cases (2019)	Case report	Report of a male child with severe hepatic dysfunction at an early age of 16 months. This shows that not all hepatic involvements in the disease improve with age. Hence all pediatric healthcare providers should be aware and do thorough investigation and continuous close monitoring of liver functions in children diagnosed with SDS.
Liebman, et al. [19]	Clinical Pediatrics (1979)	Case report	The case of a 15-month-old male with asymptomatic persistent liver disease. This shows obscure hepatic involvement and hence thorough hepatic screening should be done in SDS.
Savilahti, et al. [22]	Acta Paediatrica (1984)	Case report	Sixteen Finnish patients studied for 17 years with eight cases found at autopsy with cardiac failure due to lesions in left ventricle showing necrosis of the myofibers. Also, a transient heart failure was seen in one of the remaining eight patients.
Kopel, et al. [23]	Cardiology in the Young (2011)	Case report	SDS patients diagnosed at autopsy, died of cardiac failure due to dilated cardiomyopathy with pulmonary hypertension. This shows the involvement of heart in this disease is a serious complication that leads to death if left undiagnosed.
Toiviainen-Salo, et al. [26]	Journal of Pediatrics (2009)	Retrospective and cross-sectional study	Study carried out on 12 patients aged 2–37. It showed most patients with increased transaminase levels and hepatomegaly that resolved by age 5. Hepatic micro cysts were found in three middle-aged patients. Through continuous follow-up, mild cholestasis was recorded even after normal transaminase levels. This reflects a hepatic metabolism alteration in SDS.

Genetic and Molecular Basis of the disease

The SBDS gene mutation on chromosome 7 at position 7q11 is the bi-allelic genetically mutated molecule responsible for this disease. This gene is inherited in an autosomal recessive manner affecting multiple organs [1,5,27].

**Figure 1:**
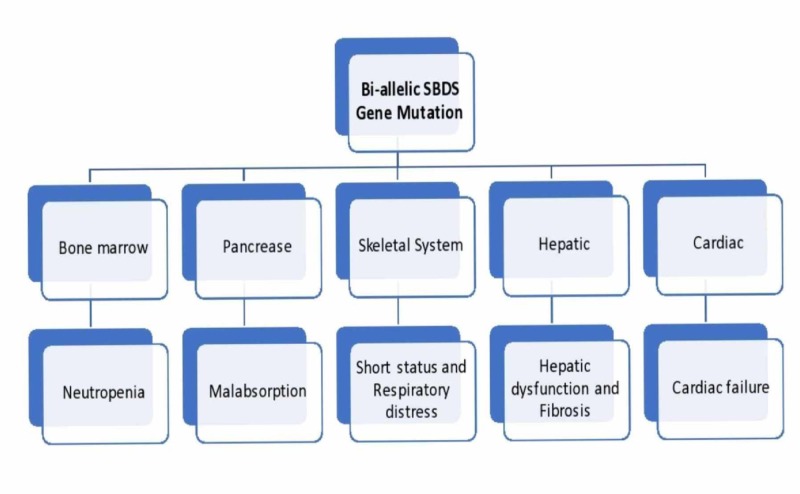
Flowchart highlighting the gene mutation and targeted organs mostly affected with their complications SBDS: Shwachman-Diamond-Bodian syndrome

The SBDS gene plays a role in the biogenesis of the ribosome; hence, the mutation in this leads to ribosomopathies [28]. Other postulations have recently been made about mutations in other genes associated with this disease and are also involved in ribosome biogenesis, such as elongation factor-like GTPase 1 (EFL1), DnaJ heat shock protein family (Hsp40) member C21 (DNAJC21), and signal recognition particle 54 (SRP54). These give SDS-like phenotypic characteristics as the SBDS gene interacts directly with EFL1 to promote the release of the Eukaryotic Translation Initiation Factor 6 (eIF6). Also, Hsp40 Member C21 interacts by stabilizing the ribosome 80S, and then the protein trafficking facilitation occurs via SRP54, and all the processes occur for ribosome maturation [10,29]. K. Austin et al. established in their study that this ribosomal biogenetic defect can lead to mitotic spindle destabilization. This results in several downstream effects in cells with a functional apoptotic mechanism such as bone marrow. This might explain the severe bone-marrow involvement in SDS patients [30]. There are also multiple organ involvements with severe complications. (Figure 1). 

Management

The management of SDS is multidisciplinary as there is no complete cure, and the patients have to be managed based on their specific symptoms. Fat-soluble vitamin supplementation and oral pancreatic enzymes are used for the exocrine pancreatic defect, blood and platelet transfusion in anemia and thrombocytopenia respectively. Prophylactic antibiotics and granulocyte-colony stimulating factor (G-CSF) are used to decrease the risk of infections in neutropenias and during complex procedures (e.g., dental procedures). Hematopoietic stem cell transplant (HSCT) is reserved for patients with severe pancytopenia, MDS and for those who develop AML, although such patients are at increased risk of hepatic and cardiac failure due to the cardio-toxic drugs used in such procedure. Neuropsychological screening and systemic function close monitoring are also done [31].

Miscellaneous

Sibship hypothesis is involved in SDS disease as it is an autosomal recessive inherited disease. Several cases among siblings have been reported. A study by Aggett et al. discusses 21 cases among whom three pairs were siblings with the disease in various stages of severity [12]

Neurocognitive impairment is also found to occur in SDS patients. In a study involving a large cohort that Kerr and colleagues conducted, which was reported by Huang and Shimamura, a systemic neuropsychological assessment carried out on 34 children from the US and Canada were compared with 13 siblings and 20 cystic fibrosis patients. It was seen that patients with this syndrome had a significant defect in intelligence, academic achievements, perceptual skills, and language abilities. They established that one in every five SDS patients met the criteria for diagnosing intellectual disability, which suggests that the SBDS gene might play an important role in neurodevelopment, and early neurocognitive assessment should be carried out to establish an early intervention [1,32].

Limitations

Although we were able to review highly valued and quality articles, we did face few barriers such as not having full access to some of the literature as they were behind a paywall. There was also an article not in English, which posed a language barrier. Also, we could not access all research engines attached to some reports as they were only available to members.

## Conclusions

After careful review, we have been able to establish the cardiac and hepatic involvement in SDS. Hepatic involvement is predominantly benign as most authors agree that the hepatic functions become normal with increasing age. However, a few articles highlighted a severe involvement of the liver by fibrosis and hepatic failure. Only a few articles acknowledged cardiac involvement, and there is no consensus as to the exact outcome of cardiac involvement in SDS. However, many authors agree that severe complications that could lead to death may arise when the heart is affected. Researchers should carry out more investigations on SDS patients to have more information on the cardiac association, reach a consensus as to the cause of this involvement and how to better manage such patients. Pediatricians should also be mindful of these associations and complications. They should carry out early screening and put in place close cardiac and hepatic monitoring of SDS patients of a very young age to appropriately manage the disease and decrease mortality.

## References

[1] Myers KC, Davies SM, Shimamura A (2013). Clinical and molecular pathophysiology of Shwachman-Diamond syndrome: an update. Hematol Oncol Clin North Am.

[2] Alves C, Fernandes JC, Sampaio S, Paiva R de MA, Calado RT (2013). Shwachman-Diamond syndrome: first molecular diagnosis in a Brazilian child. Rev Bras Hematol Hemoter.

[3] Nelson AS, Myers KC (2018). Diagnosis, treatment, and molecular pathology of Shwachman-Diamond syndrome. Hematol Oncol Clin North Am.

[4] Calado RT, Graf SA, Wilkerson KL (2007). Mutations in the SBDS gene in acquired aplastic anemia. Blood.

[5] Burroughs L, Woolfrey A, Shimamura A (2009). Shwachman-Diamond syndrome: a review of the clinical presentation, molecular pathogenesis, diagnosis, and treatment. Hematol Oncol Clin North Am.

[6] Hassan F, Byersdorfer C, Nasr SZ (2010). Severe Shwachman-Diamond syndrome and associated CF carrier mutations. Respir Med CME.

[7] Bucciol G, Cassiman D, Roskams T (2020). Liver transplantation for very severe hepatopulmonary syndrome due to vitamin A-induced chronic liver disease in a patient with Shwachman-Diamond syndrome. Orphanet J Rare Dis.

[8] Ryan TD, Jefferies JL, Chin C (2015). Abnormal circumferential strain measured by echocardiography is present in patients with Shwachman-Diamond syndrome despite normal shortening fraction. Pediatr Blood Cancer.

[9] Revert Lázaro F, Pérez Monjardín E, Pérez AP (2006). Hypertransaminasemia as a manifestation of Shwachman-Diamond syndrome. (Article in Spanish). An Pediatr (Barc).

[10] Bezzerri V, Cipolli M (2019). Shwachman-Diamond syndrome: molecular mechanisms and current perspectives. Mol Diagn Ther.

[11] Graham AR, Walson PD, Paplanus SH, Payne CM (1980). Testicular fibrosis and cardiomegaly in Shwachman’s syndrome. Arch Pathol Lab Med.

[12] Aggett PJ, Cavanagh NP, Matthew DJ, Pincott JR, Sutcliffe J, Harries JT (1980). Shwachman’s syndrome: A review of 21 cases. Arch Dis Child.

[13] Ong SY, Li ST, Wong GC, Ho AYL, Nagarajan C, Ngeow J (2020). Delayed diagnosis of Shwachman diamond syndrome with short telomeres and a review of cases in Asia. Leuk Res Rep.

[14] Sakamoto KM, Shimamura A, Davies SM (2010). Congenital disorders of ribosome biogenesis and bone marrow failure. Biol Blood Marrow Transplant.

[15] Kuijpers TW, Nannenberg E, Alders M, Bredius R, Hennekam RC (2004). Congenital aplastic anemia caused by mutations in the SBDS gene: a rare presentation of Shwachman-Diamond syndrome. Pediatrics.

[16] Nishimura G, Nakashima E, Hirose Y (2007). The Shwachman-Bodian-Diamond syndrome gene mutations cause a neonatal form of spondylometaphysial dysplasia (SMD) resembling SMD Sedaghatian type. J Med Genet.

[17] Keereweer S, Appel IM, Hoeve LJ (2012). Subglottic stenosis in Shwachman-Diamond syndrome - Is there a link?. Int J Pediatr Otorhinolaryngol.

[18] Camacho SM, McLoughlin L, Nowicki MJ (2020). Cirrhosis complicating Shwachman-Diamond syndrome: a case report. World J Clin Cases.

[19] Liebman WM, Rosental E, Hirschberger M, Thaler MM (1979). Shwachman-Diamond syndrome and chronic liver Disease. Clin Pediatr (Phila).

[20] Brueton MJ, Mavromichalis J, Goodchild MC, Anderson CM (1977). Hepatic dysfunction in association with pancreatic insufficiency and cyclical neutropenia: Shwachman Diamond syndrome. Arch Dis Child.

[21] Ritchie DS, Angus PW, Bhathal PS, Grigg AP (2002). Liver failure complicating non-alcoholic steatohepatitis following allogeneic bone marrow transplantation for Shwachman-Diamond syndrome. Bone Marrow Transplant.

[22] Savilahti E, Rapola J (1984). Frequent myocardial lesions in Shwachman’s Syndrome: eight fatal cases among 16 Finnish patients. Acta Paediatr Scand.

[23] Kopel L, Gutierrez PS, Lage SG (2011). Dilated cardiomyopathy in a case of Shwachman-Diamond syndrome. Cardiol Young.

[24] Le Gloan L, Blin N, Langlard JM (2014). Atrioventicular septal defect in a case of Shwachman-Diamond syndrome. Cardiol Young.

[25] Toiviainen-Salo S, Pitkänen O, Holmström M (2008). Myocardial function in patients with Shwachman-Diamond syndrome: aspects to consider before stem cell transplantation. Pediatr Blood Cancer.

[26] Toiviainen-Salo S, Durie PR, Numminen K, Heikkilä P, Marttinen E, Savilahti E, Mäkitie O (2009). The natural history of Shwachman-Diamond syndrome-associated liver disease from childhood to adulthood. J Pediatr.

[27] Farooqui SM, Zulfiqar H, Aziz M (2019). Shwachman-Diamond Syndrome. http://www.ncbi.nlm.nih.gov/pubmed/29939643.

[28] Provost E, Ashar F, Parsons M, Leach S (2011). Shwachman Diamond Syndrome is a p53-independent ribosomopathy. Dev Biol.

[29] Finch AJ, Hilcenko C, Basse N (2011). Uncoupling of GTP hydrolysis from eIF6 release on the ribosome causes Shwachman-Diamond syndrome. Genes Dev.

[30] Austin KM, Gupta ML Jr, Coats SA (2008). Mitotic spindle destabilization and genomic instability in Shwachman-Diamond syndrome. J Clin Invest.

[31] Nelson A, Myers K (2008). Shwachman-Diamond Syndrome. GeneReviews.

[32] Huang JN, Shimamura A (2011). Clinical spectrum and molecular pathophysiology of Shwachman-Diamond syndrome. Curr Opin Hematol.

